# Supplementary figures and images for: Mutualism in disguise: A mosquito parasite with mixed transmission mode displays mutualistic traits promoting oogenesis
Source: PLoS Pathog. 2026 Mar 9;22(3):e1014034. doi: 10.1371/journal.ppat.1014034 (PMC13008248; doi:10.1371/journal.ppat.1014034)

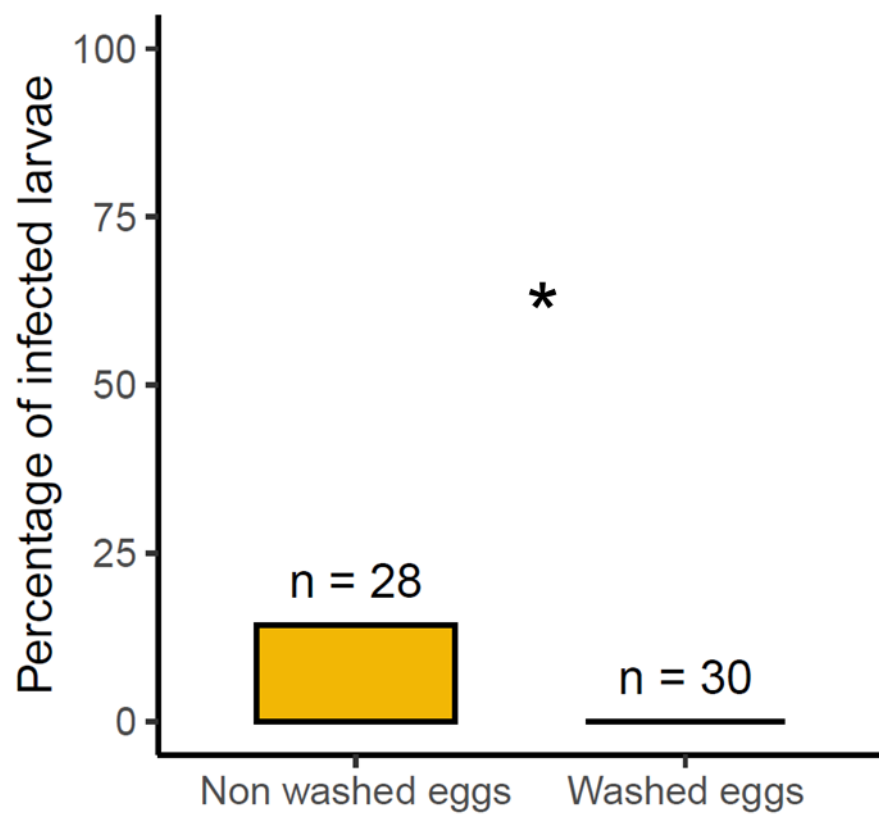

Supplement: S1 Fig — The proportion of infected L4 larvae was estimated after hatching from eggs transferred directly into fresh water (Non washed eggs) or from washed eggs transferred into fresh water (Washed eggs). The number of screened larvae is indicated for each condition (n). Statistical significance was assessed using Fisher’s exact test (p = 0.048). (PDF) [file ppat.1014034.s001.pdf]

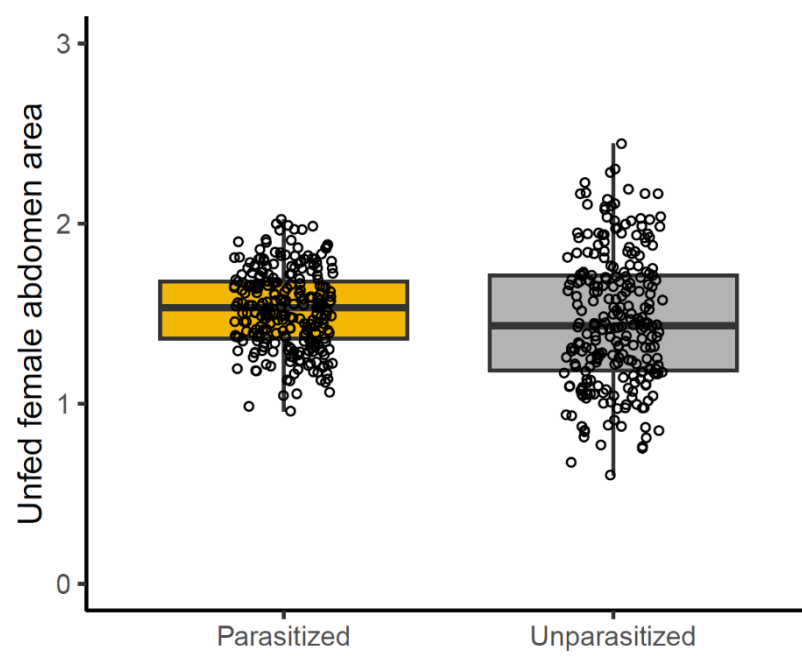

Supplement: S2 Fig — The area was measured on unfed females from parasitized and unparasitized mosquito lines. The value is expressed in mm2. Welch’s t-test p-value = 0.0253. (PDF) [file ppat.1014034.s002.pdf]

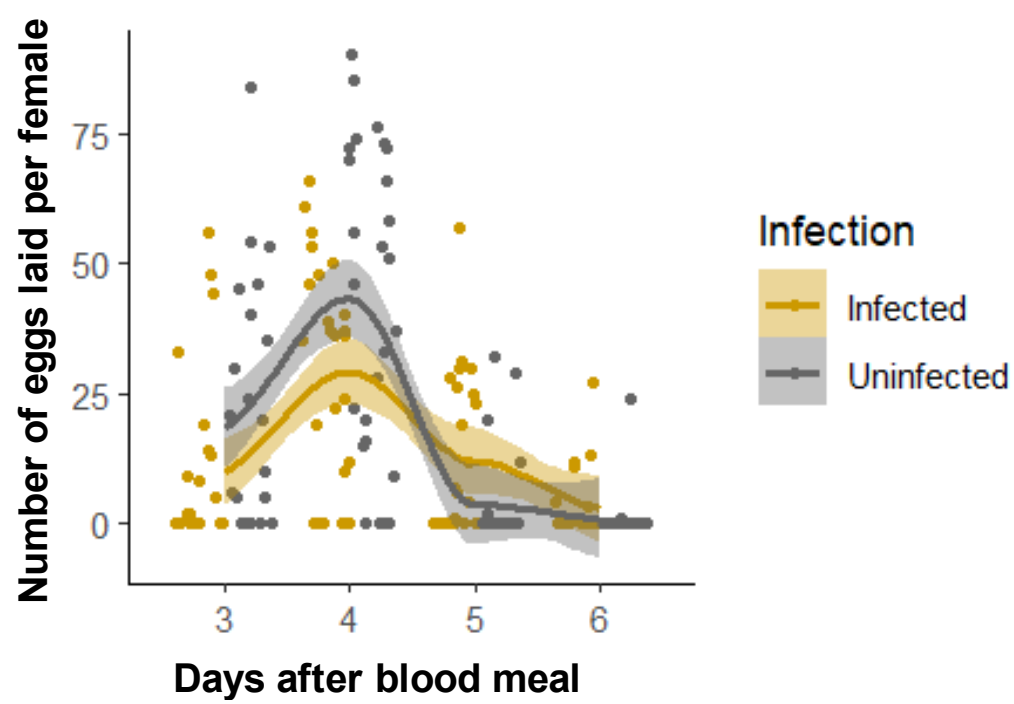

Supplement: S3 Fig — The number of eggs laid by each female was recorded daily and reported for each day of the oviposition period. (PDF) [file ppat.1014034.s003.pdf]

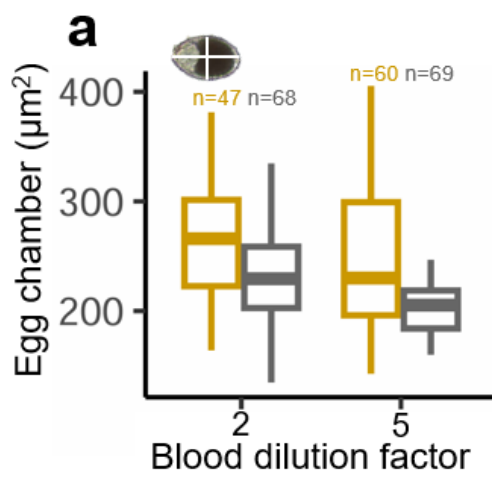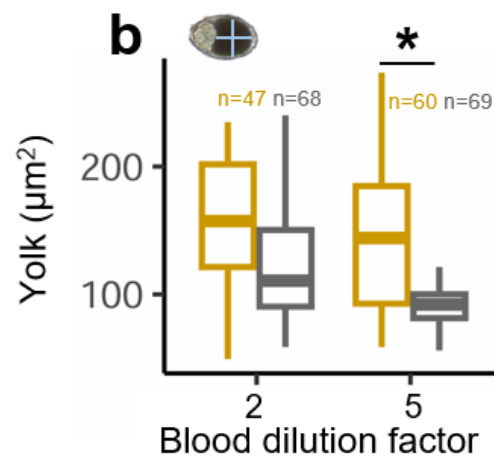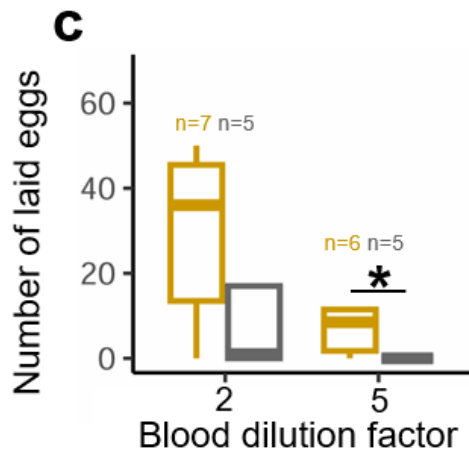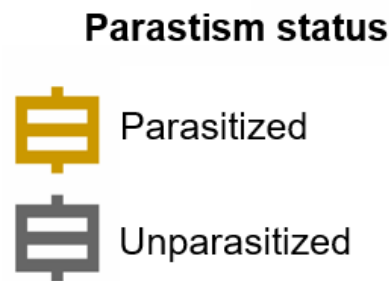

Supplement: S4 Fig — (a) The follicle primary chamber and (b) yolk areas are reported for parasitized and unparasitized females 1DABM female mosquitoes using 1/2 or 1/5 diluted blood. (c) The number of eggs laid in each of those conditions was reported. Asterisks represent significant pairwise differences at a threshold of p ≤ 0.05 from (b) post hoc Tukey HSD and (c) Wilcoxon rank-sum tests. (PDF) [file ppat.1014034.s004.pdf]

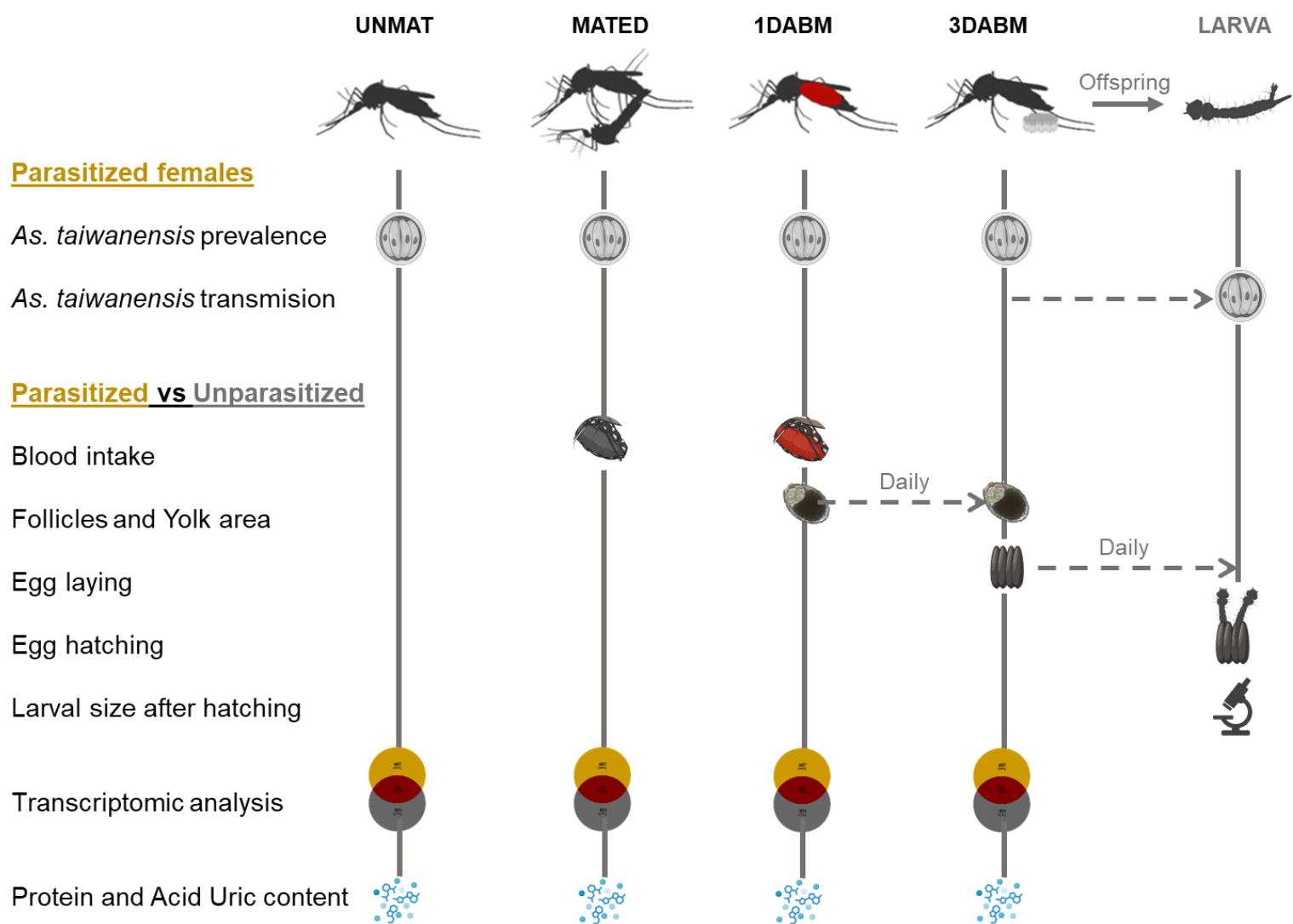

Supplement: S5 Fig — Ascogregarina taiwanensis prevalence was assessed in Ae. albopictus females at four life stages: unmated (UNMAT), mated (MATED), one day after blood meal (1DABM), and three days after blood meal (3DABM). Parasite transmission to offspring was evaluated via water and egg smearing. Blood intake was quantified by measuring abdomen width before and after feeding. Oogenesis was monitored by tracking primary follicle and yolk development over time. Egg-laying dynamics and larval size were recorded. Comparative transcriptomic analyses were performed to assess both parasite gene expression and its impact on the mosquito transcriptome. Protein content, uric acid levels, and uricase activity were also measured at each stage. Biorender was used to generate the figure (Licence number TW29FC9MJM). Created in BioRender. Girard, M. (2026) https://BioRender.com/lyuz2tc. (PDF) [file ppat.1014034.s005.pdf]
