# Supplementary material for: Mutualism in disguise: A mosquito parasite with mixed transmission mode displays mutualistic traits promoting oogenesis
Source: PLoS Pathog. 2026 Mar 9;22(3):e1014034. doi: 10.1371/journal.ppat.1014034 (PMC13008248; doi:10.1371/journal.ppat.1014034)
Supplement: Attachment — Submitted filename: Response to Reviewers Plos P_final.pdf [file ppat.1014034.s009.pdf]

Response to Reviewers

Manuscript title: *Mutualism in disguise: a mosquito parasite with mixed transmission mode displays mutualistic traits promoting oogenesis*

Journal: PLOS Pathogens

1. General Response

We thank the Editor and the Reviewers for their careful evaluation of our manuscript and for their constructive and insightful comments. We are pleased that both reviewers found the study timely, well executed, and of broad interest.

In response to the comments, we have revised the manuscript extensively to (i) clarify the genetic background and homogeneity of both host and parasite lines, (ii) provide additional methodological details regarding parasite exposure and infection status, (iii) refine our interpretation of transmission modes, particularly regarding egg smearing, and (iv) narrow and clarify our conclusions concerning mutualistic traits. We have also addressed all minor editorial and presentation comments and complied with all journal requirements.

Below, we respond point-by-point to all comments. Reviewer comments are reproduced in *italics*, followed by our responses in blue. Line numbers refer to the revised manuscript.

2. Editorial Comments

Editor comment: Genetic homogeneity of host and parasite lines

The revision must clarify the genetic homogeneity of both host and parasite lines, as this is essential for interpreting transmission patterns.

Response:
We agree that controlling genetic homogeneity is essential for interpreting transmission routes and evolutionary dynamics. This point has now been clarified (See our answer to Reviewer 1).

Editor comment: Verification of parasitization status and egg smearing

The authors should verify the parasitization status of females and provide evidence if they wish to maintain the claim regarding egg-smearing as a vertical transmission route.

Response:

The **parasitization rate in females exceeds 75% in all conditions within the parasitized population** (Fig. 1B; revised Methods) and **decreases with age due to oocyst release and the lack of parasite multiplication in adults**. In contrast, **parasitization in the unparasitized population is 0%**, as reported in the revised Methods (lines 406-409, 426-427) and in our previous study (Martin *et al.*, 2022, *Parasites & Vectors*).

“Additionally, a subset of 20 parasitized and 20 unparasitized non-fed females was examined during the experiment. The infection rate was 80% (16/20) in the parasitized population, with a mean parasite intensity of 13,125 ± 8,139 oocysts per individual, and 0% (0/20) in the unparasitized population.”

“As a control, ten females from the unparasitized population were examined for infection at the same life stages as parasitized individuals to confirm the absence of oocysts.”

Because **assessing parasitic status is destructive and because part of the individuals release the parasite along their life**, systematic verification in all conditions is **not possible and/or could be biased due to sherry picking** (e.g. selection of individuals that would be less prompt to release the parasite oocysts along their life).

However, parasite transmission rates in our assays—particularly via the most efficient route (water)—were consistent with those observed in adults, and parasite transcripts were detected in all parasitized RNA-seq samples.

Finally, the potential inclusion of rare, truly unparasitized individuals in our analyses—which cannot be entirely ruled out—would bias our results toward **smaller effect sizes rather than producing false positive effects**. This clarification has now been added to the method section (Lines 409-411).

“Because infection prevalence in the parasitized line does not reach 100%, the effects attributed to the parasite in our results are likely conservative and may therefore be underestimated.”

Egg smearing is a minor transmission route, as only 12.2% of larvae became infected when eggs were hatched in unparasitized fresh water. However, we agree that oocysts may be detached when eggs are immersed. To address the reviewer’s concern, we **conducted an additional experiment** in which eggs from parasitized females were either rinsed or not before hatching in fresh water. Infection prevalence was 14.3% in the non-rinsed condition and 0% after rinsing. **This demonstrates that egg smearing occurs but that parasites are easily removed from the egg surface during flooding**. We have now corrected our manuscript accordingly (see lines 438-445, 139-143, Fig. S1).

“Because egg smearing appeared weak, a second experiment was conducted to determine whether oocysts were firmly attached to the eggs. While larval infection prevalence reached 14.3% in larvae emerging from directly flooded eggs, no infected larvae were observed from rinsed eggs, suggesting that the parasite can be easily detached when eggs are immersed in water (Fig. S1).”

“To determine whether oocysts associated with eggs were firmly attached or could be easily detached when eggs are flooded, an additional control experiment was performed. Eggs laid by parasitized females were collected from a large cage containing approximately 5,000 individuals. One hundred eggs were directly hatched in 50 mL of unparasitized freshwater. Another set of 100 eggs was rinsed three times with fresh water and then allowed to hatch in a new container containing 50 mL of unparasitized freshwater. The prevalence of trophozoites in larvae was assessed in 28 individuals from the first condition and 30 individuals from the second condition.”

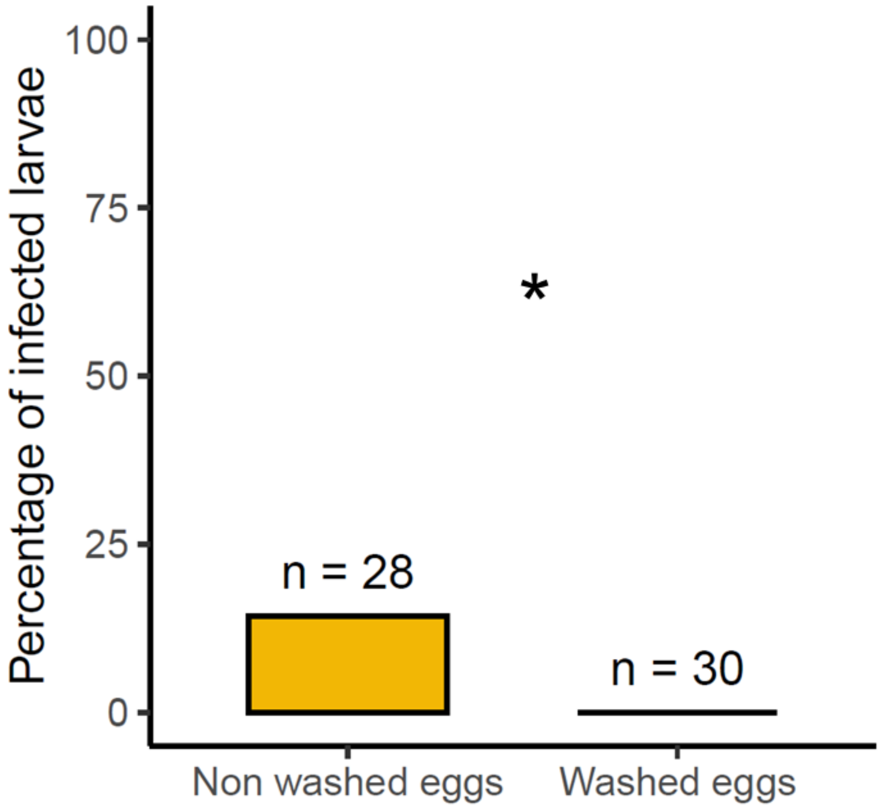

Figure S1. Egg smearing validation essay. The proportion of infected L4 larvae was estimated after hatching from eggs transferred directly into fresh water (Non washed eggs) or from washed eggs transferred into fresh water (Washed eggs). The number of screened larvae is indicated for each condition (n). Statistical significance was assessed using Fisher's exact test (p = 0.048).

3. Reviewer #1

Major comment: Importance of genetic homogeneity vs transmission mode

The manuscript lacks information on the genetic structure of hosts and parasites, which is more important than the mechanical mode of transmission.

Response:

General response

We thank the reviewer for these insightful and thought-provoking conceptual comments on the eco-evolutionary dynamics of parasite virulence and mutualism. We fully agree that our initial version placed too much emphasis on transmission mode *per se*, while insufficiently considering the role of host–parasite adaptation and genetic context in shaping interaction outcomes.

Ascogregarina taiwanensis is indeed a highly host-specific parasite, and local adaptation has previously been suggested (e.g. Tseng et al., *Journal of Medical Entomology*). Moreover, we and others have shown that *Aedes albopictus* populations from France exhibit low genetic diversity and high levels of inbreeding (e.g. Minard et al., 2015, *Frontiers in Microbiology*; Goubert et al., 2017, *Molecular Ecology*), conditions that may facilitate stable physiological coupling between hosts and their associated parasites. At present, however, gregarine genetic diversity remains poorly characterized at a resolution sufficient to formally investigate parasite adaptation. Based on our previous work (Girard et al., 2024, *PNAS Nexus*), three main *Ascogregarina* operational taxonomic units occur at the global scale, with a single genotype (OTU1) dominating French populations.

Importantly, the present study relies on a single laboratory population reared either with or without its native parasite, and should therefore be viewed as a proof of concept. We fully acknowledge that our results cannot yet be extrapolated to other mosquito populations or to natural ecological conditions, where both host and parasite genetic diversity, as well as environmental heterogeneity, are likely to modulate interaction outcomes. Addressing these dimensions will require substantial additional work over the coming years.

Nevertheless, we believe that our findings provide a valuable first step toward understanding how physiological coupling during reproduction, in a context of strong host specificity and limited genetic diversity, can generate traits consistent with mutualism despite the persistence of parasitic features. In response to the reviewer's comments, we have revised the Introduction, Methods and Discussion to better acknowledge the importance of host–parasite adaptation, genetic context, and the potential role of compensatory host responses, and to temper any overly deterministic interpretation of transmission mode alone.

Detailed response

(i) We agree that controlling genetic homogeneity is essential for interpreting transmission routes and evolutionary dynamics. We believe this concern arose from insufficient description of how the parasitized and unparasitized mosquito lines were generated and maintained. Importantly, **both lines originate from the same focal mosquito population**, and the unparasitized line was directly derived from the parasitized line. Thus, host genetic background is shared between treatments.

We have now clarified the origin and maintenance of both host and parasite lines in the Methods (lines 389–409):

"Parasitized and unparasitized Aedes albopictus lines originated from a hybrid laboratory population derived from two French natural populations sampled in Villeurbanne (N: 45°46′18.990″, E: 4°53′24.615″) and Pierre-Bénite (N: 45°42′11.534″, E: 4°49′28.743″) in 2017, following a previously described protocol [21,71]. Briefly, this population was maintained in large cages (~5,000 individuals). To establish the parasitized line, we applied conditions favoring parasite transmission: (i) water containers were kept inside the cage during oviposition, (ii) larvae were reared in this same water, and (iii) during the first ten generations, ~100 parasitized adults were crushed and added to the water to reseed the next generation and ensure efficient parasite transmission. Parasite presence was verified by diagnostic PCR and microscopy as previously described [71]. To generate the unparasitized line, ~10,000 eggs originating from the parasitized line were used, and parasite transmission was deliberately disrupted. Specifically: (i) during the first generation, egg surfaces were rinsed and treated with an antiparasitic compound effective against gregarines (griseofulvin, 1 mg mL^{-1}); (ii) at each generation, eggs were systematically removed from the water container; and (iii) transferred to fresh water. Thus, both lines share the same genetic background but differ in infection status and were each maintained at ~5,000 individuals. *As. taiwanensis* presence was monitored at each generation (F_{46}) by examining crushed pools of 25 adults and samples of rearing water under ×400 magnification using an optical microscope (Leica). Additionally, a subset of 20 parasitized and 20 unparasitized non-fed females was examined during the experiment. The infection rate was 80% (16/20) in the parasitized population, with a mean parasite intensity of 13,125 ± 8,139 oocysts per individual, and 0% (0/20) in the unparasitized population."

(ii) Together, these procedures ensure that the parasitized and unparasitized lines differ in infection status only, while sharing the same genetic background and being maintained under identical demographic conditions.

In addition, previous experiments demonstrated that ***As. taiwanensis* can efficiently recolonize the unparasitized population**, indicating no apparent loss of parasite infectivity or adaptation. This point is now explicitly stated in the manuscript (Lines 411-412):

"Previous work demonstrated that *As. taiwanensis* can efficiently recolonize unparasitized populations, indicating no apparent loss of adaptation [71]."

(iii) We have **revised the Abstract, Introduction and Discussion** to clarify that transmission mode alone does not determine mutualistic outcomes, and to emphasize the role of host–parasite adaptation and genetic homogeneity in shaping the observed phenotypes (Lines 17-18, 28-30, 57-59, 76-79, 83-86, 92-96, 276-279, 289-294, 361-368, 375-379).

"Mutualistic traits are frequently associated with vertically transmitted symbionts, in part because repeated interactions can align host and symbiont fitness."

"Together, our results illustrate how physiological coupling during reproduction, combined with mother-biased transmission, can generate mutualistic traits within an interaction that also retains parasitic features, blurring the boundary between parasitism and mutualism."

“Interdependency between partners is influenced by the mode of transmission, but also by the degree of reciprocal adaptation and the genetic context in which interactions occur; vertical transmission often reinforces such dependency by aligning host and symbiont fitness [4–6].”

“In contrast, horizontal transmission can decouple symbiont fitness from that of the host, potentially favoring parasitic strategies, although such outcomes depend strongly on host–parasite adaptation and genetic homogeneity within interacting populations [1,14].”

“Together, empirical studies and controlled experiments demonstrate that vertical transmission often reduces microbial virulence and may promote the emergence of mutualistic traits, particularly when host and symbiont experience repeated interactions and opportunities for reciprocal adaptation [18,19].”

“This parasite negatively affects host survival and reproduction under nutrient-deficient conditions [22] or when infection loads are high [23]. The outcome of this interaction is further shaped by genotype-by-genotype effects between host and parasite [24,25], and *As. taiwanensis* has been suggested to contribute to the regulation of mosquito populations [26].”

“However, increasing evidence suggests that such outcomes depend not only on transmission mode *per se*, but also on the degree of host–parasite adaptation and the genetic homogeneity of interacting populations [32–35]. The pseudo-vertical transmission of *Ascogregarina* species, however, remains poorly characterized [30].”

“Rather than acting as a primary driver of mutualism, this transmission ecology may reinforce stable host–parasite associations in systems where host and parasite are already well adapted to one another.

Consistent with this view, our findings indicate that *As. taiwanensis* infection is associated with improved reproductive performance in *Ae. albopictus*. Parasitized females produced larger progeny and sustained oogenesis even under limited blood resources.”

“This study was conducted using a single laboratory line of *Ae. albopictus*, either infected or uninfected with its native parasite assemblage. Genetic diversity in this line was likely reduced through long-term laboratory maintenance, although this was not directly measured. Both mosquito and parasite field populations appear to be locally structured [21,66], while also exhibiting genetic structuring at the global scale [64,21,65]. Genotype × genotype interactions have been shown to influence parasite infection and proliferation [24]. Controlling mosquito and parasite genetic diversity could therefore help disentangle the flexibility of these interaction outcomes and should be addressed in future studies.”

“In summary, our study highlights the complexity of the interaction between *Ae. albopictus* and *As. taiwanensis*, revealing how host physiological responses to infection can offset parasite-associated costs and generate outcomes consistent with mutualistic traits under specific conditions. These traits should be further investigated in relation to other mosquito life-history traits and under more ecologically realistic conditions.”

4. **Reviewer #2**

Major comment: Are all "parasitized" females truly infected?

It is unclear whether mosquitoes labeled as parasitized were all indeed parasitized.

Response:
We agree that exposure does not guarantee infection in all individuals. We cannot estimate the parasite infection in all individuals since the method is destructive and the parasite is released by adults throughout their life. Furthermore, it is not possible to standardize the parasite intensity or prevalence in emerging adults. Even though we know from our multiple studies that it is relatively stable and approximate the value we observed in most field populations *e.g.* Girard et al., 2024 – PNAS Nexus. **As mentioned by the reviewer the parasitized line shows >75% infection rate in non-fed female individuals and we have now reported its intensity** which is 13,125 ± 8,139 oocysts per individual (see Fig. 1B and revised Methods lines 406-409). **Individuals labeled as parasitized are from the parasitized mosquito line.**

"Additionally, a subset of 20 parasitized and 20 unparasitized non-fed females was examined during the experiment. The infection rate was 80% (16/20) in the parasitized population, with a mean parasite intensity of 13,125 ± 8,139 oocysts per individual, and 0% (0/20) in the unparasitized population."

Importantly, any misclassification (which should be <25%) would increase IQDs as mentioned by the reviewer and **bias our results toward underestimating parasite effects rather than generating spurious positive effects**. We now explicitly mention this point in the methods (lines 409-411).

"Because infection prevalence in the parasitized line does not reach 100%, the effects attributed to the parasite in our results are likely conservative and may therefore be underestimated."

Major comment: What is the exposure level?

It is unclear from the methods what the exposure level that was used here was.

Response

We only seeded the population during the first generations with crushed bodies of ~100 individuals from the previous generation (we used old individuals from the previous generation to do so, and it corresponds to ~$2.56 \pm 2.14 \times 10^3$ oocysts/mL). At the time we conducted our experiment, **the transmission was not due to an active seeding of the parasite in the population, but we rather enable mosquitoes to transmit the parasite to next generations through water containers** in large cages of 5000 individuals. This is now clearly stated in the revised method version (Lines 392-398).

"Briefly, this population was maintained in large cages (~5,000 individuals). To establish the parasitized line, we applied conditions favoring parasite transmission: (i) water containers were kept inside the cage during oviposition, (ii) larvae were reared in this same water, and (iii) during the first generations, ~100 parasitized adults were crushed and added to the water to reseed the next generation and ensure efficient parasite transmission. Parasite presence was verified by diagnostic PCR and microscopy as previously described [71]."

Major comment: Egg smearing as vertical transmission

Egg smearing does not ensure mother-to-offspring transmission and may infect unrelated larvae.

Response:
We agree with this assessment. As previously mentioned, **maternal transmission to larvae** occurs predominantly **through water rather than via egg smearing**. Accordingly, throughout the manuscript **we now refer to egg smearing or the release of oocysts into water during oviposition as *pseudo-vertical transmission* or *maternal transmission bias***, rather than strict vertical transmission. We also explicitly acknowledge that oocysts deposited on eggs or within water containers may indiscriminately infect unrelated larvae once released into the environment (Introduction and Discussion, lines 106-109 and 284-289).

"Several studies have also suggested a potential pseudo-vertical transmission (i.e. maternal transmission bias toward post hatch symbiont acquisition) via egg smearing from females to their offspring [23,30], although this has yet to be clearly demonstrated."

"These oocysts efficiently infect the progeny within the water containers. We therefore suggest that oocyst transmission by females is biased toward increasing the likelihood of transmission to their own offspring, although larvae from other females sharing the same breeding site may also become infected. The relative contributions of horizontal and vertical transmission nevertheless remain to be determined"

Furthermore, in our study, egg smearing is a minor transmission route, as only 12.2% of larvae became infected when eggs were hatched in unparasitized fresh water and oocysts may also be detached when eggs are immersed. To address the reviewer's concern, we **conducted an additional experiment** in which eggs from parasitized females were either rinsed or not before hatching in fresh water. Infection prevalence was 14.3% in the non-rinsed condition and 0% after rinsing. **This demonstrates that egg smearing occurs but that parasites are easily removed from the egg surface during flooding**. We have now corrected our manuscript accordingly (see lines 139-143, 438-445, Fig. S1).

"Because egg smearing appeared weak, a second experiment was conducted to determine whether oocysts were firmly attached to the eggs. While larval infection prevalence reached 14.3% in larvae emerging from directly flooded eggs, no infected larvae were observed from rinsed eggs, suggesting that the parasite can be easily detached when eggs are immersed in water (Fig. S1)."

"To determine whether oocysts associated with eggs were firmly attached or could be easily detached when eggs are flooded, an additional control experiment was performed. Eggs laid by parasitized females were collected from a large cage containing approximately 5,000 individuals. One hundred eggs were directly hatched in 50 mL of unparasitized freshwater. Another set of 100 eggs was rinsed three times with fresh water and then allowed to hatch in a new container containing 50 mL of unparasitized freshwater. The prevalence of trophozoites in larvae was assessed in 28 individuals from the first condition and 30 individuals from the second condition."

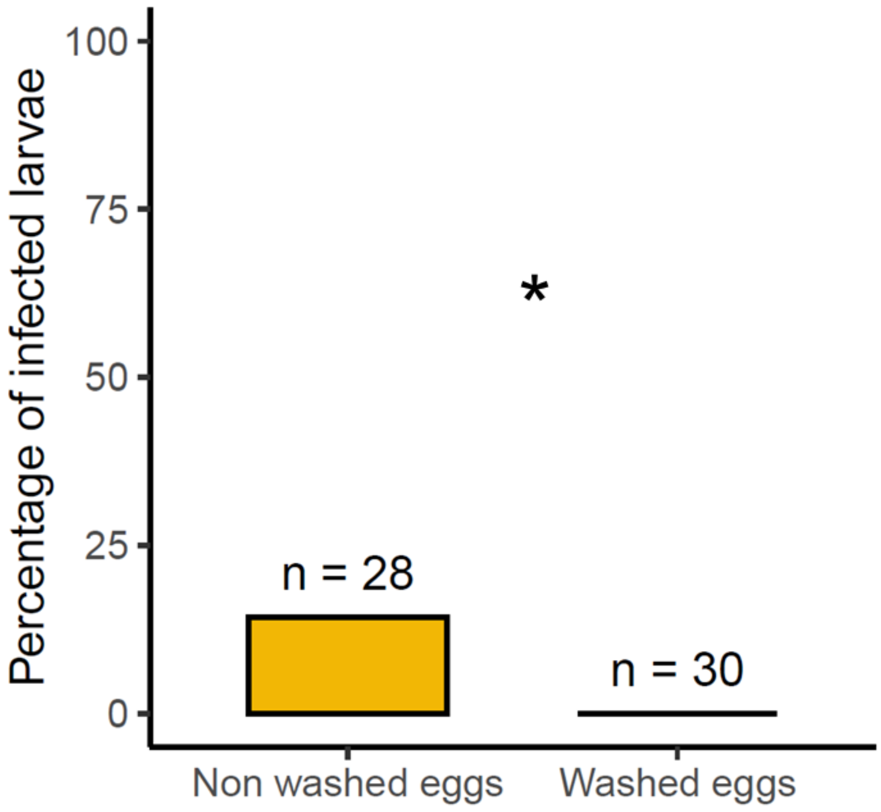

Figure S1. Egg smearing validation essay. The proportion of infected L4 larvae was estimated after hatching from eggs transferred directly into fresh water (Non washed eggs) or from washed eggs transferred into fresh water (Washed eggs). The number of screened larvae is indicated for each condition (n). Statistical significance was assessed using Fisher's exact test ($p = 0.048$).

Oviposition timing vs duration

The data indicate delayed oviposition rather than extended oviposition.

Response:
We understand the reviewer's suggestion, which may have been guided by the figure presenting the cumulative number of eggs. To further clarify this point, **we provide below an additional representation showing egg-laying dynamics** (mean number of eggs laid per females per day) as recommended by the reviewer. This figure indicates that, in all populations, the maximum number of eggs laid per female is reached at 4 days post blood meal; importantly, **this maximum would have been shifted if egg laying would have been delayed**.

We initially considered combining this dynamics figure with another panel showing the total number of eggs laid per female. However, after discussion, we concluded that the cumulative representation is preferable, as it conveys both the timing and the total egg production while limiting the number of figures in the panel. Nevertheless, we have now added the egg laying dynamic into the supplementary material section.

Finally, we noticed that some text edits introduced during manuscript revision altered the original meaning of the sentence, rendering it inconsistent with the data. This may have

contributed to a misunderstanding of the results. The paragraph has now been corrected (lines 156-163, Fig. S3).

"Egg laying was delayed in parasitized females. In both groups, most eggs were laid at 4 days after blood meal (DABM), accounting for 72.6% and 93.0% of eggs in parasitized and unparasitized females, respectively (Fig. 2e; Fig. S3). However, a temporal delay was evident, as parasitized females laid significantly fewer eggs at both 3 and 4 DABM (3 DABM: $z = -2.23$, $p = 0.026$*; 4 DABM: $z = -2.14$, $p = 0.032$*). The total number of eggs laid did not differ significantly between groups ($z = -1.46$, $p = 0.144$), although it was slightly lower in parasitized females. Hatching success was comparable between groups, regardless of whether eggs were fresh or stored (Fig. 2f)."

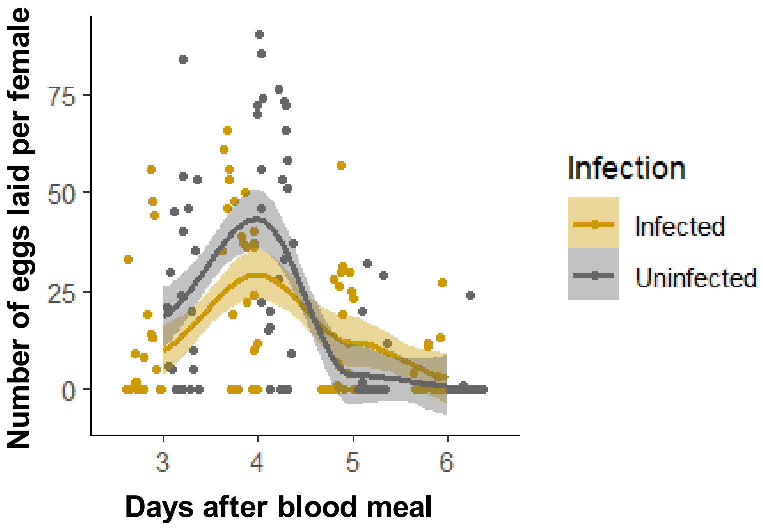

Figure S3. Dynamics of egg laying. The number of eggs laid by each female was recorded daily and reported for each day of the oviposition period.

Mutualism claims

The authors do not measure sufficient fitness components to conclude mutualism.

Response:
We agree and have **revised the manuscript to consistently refer to "mutualistic traits" rather than a definitive mutualism**. We now explicitly state that our conclusions are limited to the fitness components measured under laboratory conditions and that additional work will be required to assess net fitness consequences under natural or competitive conditions (e.g. lines 314-315, 351, 375-381).

"Our study further supports the presence of host physiological responses to *As. taiwanensis* infection that generate outcomes consistent with mutualistic traits […]"

"While our data suggest the existence of mutualistic traits […]"

“In summary, our study highlights the complexity of the interaction between *Ae. albopictus* and *As. taiwanensis*, revealing how host physiological responses to infection can offset parasite-associated costs and generate outcomes consistent with mutualistic traits under specific conditions. These traits should be further investigated in relation to other mosquito life-history traits and under more ecologically realistic conditions.”

Section by section feedback

L71: There is some evidence that other characteristics than just nutrient amounts may impact host fitness - although in another species of Ascogregarina, Sulaiman (1992) demonstrated that some strains are more pathogenic to some populations of host, which may indicate a degree of adaptation of different populations. Tseng (2017) provides further evidence of this with As. taiwanensis and Ae. albopictus due to the apparent "escape" from parasitism by hybrid Ae. albopictus. In the field, various authors report quite varying infection intensity (sometimes seasonal), and Soghigian and Livdahl (2021) shows some degree of population regulation. While field larvae may be under nutrient defecient conditions broadly, perhaps those are the more natural condition of hosts.

Response:

We thank the reviewer for her/his constructive input on the Introduction. The manuscript has been revised accordingly (Lines 89-101).

“The Asian tiger mosquito, *Aedes albopictus*, is the most invasive vector of vertebrate pathogens, and its global spread is a major concern for public health policies [20]. Field populations of *Ae. albopictus* are commonly and heavily parasitized by *Ascogregarina taiwanensis*, a low-virulence Apicomplexan entomoparasite [21]. This parasite negatively affects host survival and reproduction under nutrient-deficient conditions [22] or when infection loads are high [23]. The outcome of this interaction is further shaped by genotype-by-genotype effects between host and parasite [24,25], and *As. taiwanensis* has been suggested to contribute to the regulation of mosquito populations [26]. However, it has also been suggested that *As. taiwanensis* may have contributed to the competitive success of *Ae. albopictus* over other mosquito species during the invasion of new habitats—such as breeding sites also colonized by *Ae. aegypti* [27]. This may be due to the higher virulence of *As. taiwanensis* toward *Ae. aegypti* larvae, while exerting limited impact on the fitness of its natural host [28]. Consequently, *As. taiwanensis* may have either negative or indirectly beneficial effects on *Ae. albopictus*.”

L81: Interesting distinction between horizontal and vertical here. Just a comment, rather than something to address, but anecdotally it seems container-dwelling mosquitoes will revisit the same container they emerge from to lay eggs; in this case, horizontal and vertical transmission modes end up overlapping. I am also not sure that I would agree that

Response:

This is indeed a very interesting point that needs to be further explored.

L81: Hm, the more I think about this, the less certain I am that this is indeed vertical transmission. Mosquitoes often develop in larval habitats with unrelated conspecifics, other mosquito species, and other aquatic organisms. Many of these organisms may consume the parasite material, such that it is not, strictly speaking, vertical transmission. I would instead think that egg smearing increases the probability of transmission from mother to offspring, but in the same way that defecation and/or dying on the water and/or reusing a larval habitat a female emerged from would. Do we know that e.g. egg smearing increases the chance that larvae will be infected, or increases the parasite load?

Response:

We agree that the release of oocysts, which is enhanced during oviposition, does not correspond to strict vertical transmission but rather to pseudo-vertical transmission, as oocysts can subsequently be ingested by unrelated larvae.

Importantly, these results nevertheless demonstrate that maternal transmission exists and can be efficient under laboratory conditions, when only maternal transmission is possible and in the absence of any conspecifics or other species in the container. Any conclusion regarding its relative importance under more realistic conditions, or when it co-occurs with horizontal transmission, would be speculative.

We also emphasize that our results cannot be extrapolated to parasite load or to the relative contribution of horizontal transmission. First, parasite load was difficult to quantify accurately in larvae when infections were dense and mixed with the food bolus; however, parasite presence could be reliably detected because trophozoites are large. Larvae were used because a fraction of the parasites is released at adult emergence. Second, we could not compare maternal and horizontal transmission in this study. The objective here was therefore to provide a proof of concept that maternal transmission is possible and likely occurs, as females tend to release oocysts during oviposition and these oocysts efficiently infect their progeny, primarily through the water container. This point is now discussed in the manuscript (Lines 282-288).

"Since *As. taiwanensis* is expelled from adult mosquitoes without replicating in the adult stage [29], our results support the hypothesis that relatively few parasites are released by females prior to oviposition, while oocyst release is enhanced during egg formation and oviposition. These oocysts efficiently infect the progeny within the water containers. We therefore suggest that oocyst transmission by females is biased toward increasing the likelihood of transmission to their own offspring, although larvae from other females sharing the same breeding site may also become infected. The relative contributions of horizontal and vertical transmission nevertheless remain to be determined."

L110: Did the authors observe female oviposition behavior at all? Were all females left to die in the water after oviposition? There are some interesting implications here, as at least in Ae. aegypti, females contact water during oviposition just with their feet ("tasting" it) rather than directly on it, so it is possible that egg smearing may be a primary way that a skip ovipositing species distributes Ascogregarina, unless the female was also defecating during oviposition.

Response:

Oviposition was recorded daily, and females that died during the experiment were excluded from the analysis, as now clarified in the Methods section (lines 438, 461). No dedicated behavioral experiments were conducted in this study, as oviposition behavior is the focus of ongoing and future work. Nevertheless, we now discuss this point in the manuscript, particularly the potential importance of skip oviposition. We also clarify that parasite transmission is most likely mediated through contamination of the water container (likely via female defecation) rather than through egg-smearing *per se* (see Discussion, lines 282-285, 296-301).

"Females that died were removed from the analysis"

"Since *As. taiwanensis* is expelled from adult mosquitoes without replicating in the adult stage [29], our results support the hypothesis that relatively few parasites are released by females prior to oviposition, while oocyst release is enhanced during egg formation and oviposition. These oocysts efficiently infect the progeny within the water containers."

"Additionally, parasitized females exhibited altered oviposition behavior, distributing their eggs over a longer period than unparasitized females. This trait could increase both reproductive success and parasite transmission. *Aedes albopictus* females typically distribute their eggs among multiple containers—a bet-hedging strategy known as skip oviposition [37]. Consequently, an extended egg-laying period may enhance the dispersal of both eggs and oocysts across breeding sites."

L125: Really interesting observation on the shift in egg-laying. Do the authors have any estimate on adult size for ovipositing females? Were parasitized females smaller? I wonder if this could explain this shift - may be worth mentioning, as there is evidence that Ascogregarina reduce adult size I believe.

Response:
We have now added a measurement of female abdomen surface, which is a proxy of overall female size and fecundity potential. Parasitized females exhibit a slightly larger abdomen; however, the effect size is almost negligible. We refer the reviewer to our response to the comment related to line 218 below for further details.

L128: Hm, Figure 2E certainly looks like there were fewer eggs laid by parasitized females, though I recognize the difference is not significant. The authors may want to present the average per female per day here, from the 25 females, rather than the total count.

Response:

Indeed, the number of eggs was slightly higher, and we agree that a lack of statistical significance does not imply an absence of effect. This point is now clearly stated in the manuscript (lines 160-161). In addition, a figure illustrating the egg-laying dynamics has been added to the Supplementary Data, as explained above.

“The total number of eggs laid did not differ significantly between groups ($z = -1.46$, $p = 0.144$), although it was slightly lower in parasitized females.”

L130: Given parasitized mosquitoes are generally smaller, and given that females can 'pass on' their infection, I wonder if this minor size advantage at first instar will actually translate through the life cycle.

Response:

In our study, we did not observe any major differences in adult size. Female abdomens were even slightly wider (see our response below). While the fate of progeny conditioned by parental parasitic status and parasite transmission represents an important question, it was outside the scope of M. Girard’s PhD work and therefore not addressed in the present study. This topic is being investigated by a Postdoc researcher in a separate, parallel project. Given the substantial workload for each study, it was not feasible to merge them, and the present manuscript was designed to remain focused on reproduction.

L218: This is a very interesting experimental design... But what size were females that were used here? It seems possible that if parasitized females were smaller, they may be able to make use of less bloodmeal resources than larger females, given that the bloodmeal is also used for host nutrition (albeit in a minor way).

Response:

We initially compared female abdomen width and detected no differences before or after the blood meal (Fig. 2a). However, we agree with the reviewer that this analysis does not fully address the possibility that parasitized females might be smaller overall, or that their abdomen length could differ. Unfortunately, the same samples could not be used for this purpose because the original photographs were focused on the widest part of the abdomen only.

To address this point, we therefore performed an additional analysis using a different dataset (acquired in parallel on the same populations) comprising unfed females for which full-abdomen images were available (261 parasitized and 254 unparasitized individuals). In this dataset, parasitized females exhibited a slightly larger abdomen surface. However, the effect size was extremely small (95% CI: 0.007–0.112 mm^2), and the statistical significance (Welch’s t-test, $p = 0.02$) is most likely driven by the large sample size rather than reflecting a biologically meaningful difference.

These results have now been added to the Supplementary Data, method and Results sections and are discussed in the manuscript (Lines 147-149, 450-452, Fig S2).

“Parasitized females harbor a slightly larger abdomen surface than their unparasitized conspecific (1.52±0.220mm2 vs. 1.46±0.367mm2, $t=2.23$, $p=0.0253$; Fig. S2).”

“Additionally, 261 unfed parasitized females and 254 unfed unparasitized females were photographed as x7.5 magnification, and their abdomen surface was measured in ImageJ v2.0.”

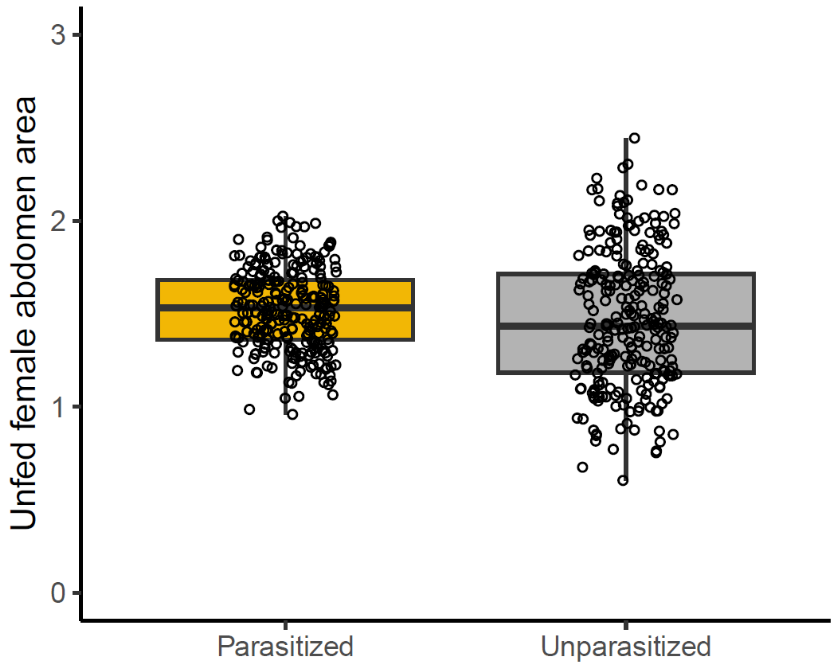

Figure S2. Abdomen area of parasitized and unparasitized females. The area was measured on unfed females from parasitized and unparasitized mosquito lines. The value is expressed in mm^2. Welch's t-test p-value = 0.0253.

L218: In addition, when looking at supplemental figures... It appears that unparasitized females still had egg development, just not oviposition.

Response:

Indeed, they develop but then get resorbed (see photos below). The females never lay them when fed on deprived blood meal.

Here is a representative image showing, in the left panel (D) developing eggs in the ovaries of parasitized females, and in the right panel (E) resorbing eggs in unparasitized females.

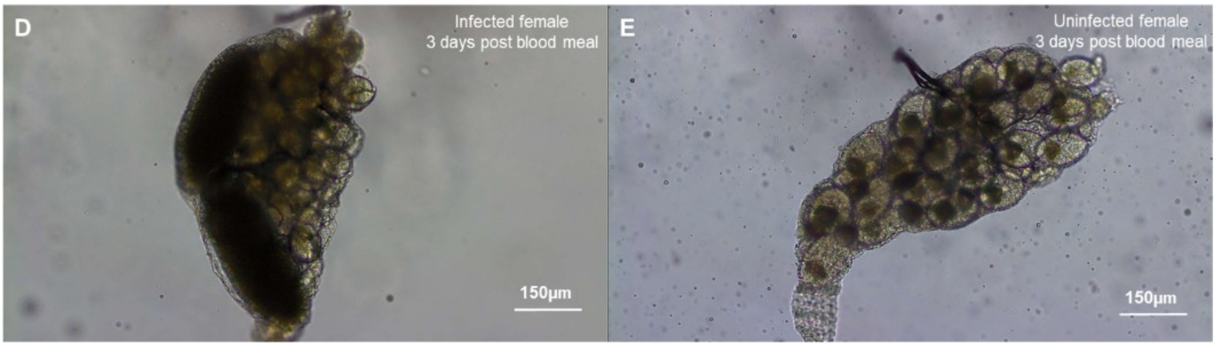

L257: I don't agree with many of these statements, based on my understanding of the author's experimental design. From what I understand, mosquitoes were transferring to oviposition environments basically starting on Day 3. Then, both sets of females oviposited primarily over a two day period; it is just that infected females took an extra day for to start ovipositing in earnest. As females were not given access to oviposition sites before this, we don't know if, e.g., unparasitized females may have begun earlier than parasitized if given the option. Since the primary pattern appears to be a shift to a day later in parasitized females, this indicates a longer time until oviposition, NOT an extended period of oviposition / more skip oviposition. As such, I do not see the pattern the authors report of an increased length of oviposition - it looks like all treatments continued to lay a small number of eggs after day 4, as both boxplots shift slightly upwards.

Response.

We hope that our response above helps to clarify this point. Based on our data, we are confident that the effect observed reflects an extension of the oviposition period rather than a delay in laying eggs. Eggs laid by each female were counted daily throughout the entire experiment. Monitoring was conducted daily from day 2 onward. This choice was based on consistent observations that mosquitoes do not lay eggs before this point, which corresponds to the minimal time required for egg development. As shown in Fig. 2c and d, eggs are not developed on day 1. In addition, numerous pilot experiments conducted for this study and previous work consistently confirmed the absence of egg laying before day 3.

In the Methods section, we indicate that the experiment was conducted for 6 days because no eggs were laid on day 7, at which point the experiment was terminated. This cutoff was based on both the present dataset and prior pilot experiments, in which egg laying was never observed beyond this time period.

We clarified this point in the method section (Lines 465-467)

"Females were transferred daily to new tubes for 6 days, during which egg numbers were recorded; no eggs were laid beyond this time window."

L272: I would limit this mutualism commentary to specifically what the authors have measured. We have no idea of how large females are, or how large parasitized larvae are in habitats with conspecifics, only at first instar. We do not know the survival of such larvae, etc. The authors have certainly demonstrated mutalistic traits; however, they have not definitively shown a mutualism as we do not know the ultimate fitness consequences of parasite infection from this study. Interestingly, there is another study with implicated mutualist traits in Ascogregarina (Soghigian et al. 2017), though it appears it is in a different species of parasite and Aedes host.

Response.

As mentioned before, we agree with the reviewer that we should not overinterpret our results and stick to the results. We are now discussing the flexibility of those interactions with the

reference suggested by the reviewer along with few others. Here is a revised version of the discussion (Lines 366-373).

"Controlling mosquito and parasite genetic diversity could therefore help disentangle the flexibility of these interaction outcomes and should be addressed in future studies. In addition, the nature of this interaction should be investigated for other fitness-related traits and under more complex conditions (*e.g.* varying ecological contexts) to better assess its consequences for mosquito fitness. Indeed, gregarine–mosquito relationships have been shown to vary with respect to behaviour, development, and survival depending on the context [23,26,67–69]. Finally, although the prevalence of *Ascogregarina taiwanensis* is high at the global scale, it varies among populations and over time [21,26,70]."

L288: This is extremely interesting, but it is unclear how oocysts would acquire glutamine while in that stage.

Response.

Unfortunately, little is known about the potential routes of transport at this stage, and we found no relevant information in the literature for Apicomplexa. This lack of knowledge is now explicitly highlighted in the Discussion (Lines 334-335).

"However, the routes of transport at this stage remain to be investigated."

L302: While skip oviposition most certainly is a valuable bet hedging strategy, this statement reads as if to suggest As. taiwanensis is triggering skip oviposition - it does not seem so. Rather, there seems to be delayed oviposition,

Response.

As shown above, the phenotype is indeed an extended oviposition period, which may increase the possibility of mosquitoes visiting more oviposition sites. We agree that this point of discussion is anyways a little bit speculative, but we think it would be valuable to keep it in order to open new perspectives for future studies.

L316: I was under the impression the study the authors cite has to do with behavioral changes in response to partial bloodmeals, not diluted bloodmeals. Moreover, adult female size seems confounding here, as it is quite possible that parasitized females were smaller than unparasitized females and able to better utilize partial bloodmeals.

Response.

As shown above, abdomen size was very similar between parasitized and unparasitized females, and was even slightly larger in parasitized females. Moreover, no difference in abdomen width was observed between groups after blood feeding. We acknowledge that partial blood meals and diluted blood meals are not strictly equivalent in terms of ingested

volume, although they are comparable in terms of resource availability. This distinction is now stated more explicitly in the manuscript (Lines 356-359).

“Notably, fecundity impairment occurred only in unparasitized females fed with diluted blood—conditions mimicking reduced resource acquisition associated with host defensive behaviour, which leads to partial feeding in nature [63].”

L337: Was only presence recorded, or was infection intensity also recorded? There appears to be dosage-related effects of As. taiwanensis from the literature (Sulaiman 1992 and others).

Response.

Parasite intensity has now been reported (lines 407-409)

“The infection rate was 80% (16/20) in the parasitized population, with a mean parasite intensity of 13,125 ± 8,139 oocysts per individual, and 0% (0/20) in the unparasitized population.”

L349: What happened to females after they finished ovipositing? Did they die in the water? Minor - it does not impact the robustness of the vertical transmission assay - but worth noting if so.

Response.

Any female that died in the water container was removed from the analysis for the oviposition dynamics and the transmission essays. This is now explicitly stated in both parts of the method section (Lines 438 and 461).

“Females that died were removed from the analysis.”

L366: How long were females with diluted blood given to oviposit?

Response.

For both experiments using either pure or diluted blood meals, female mosquitoes were allowed a full week (7 days) after the blood meal to oviposit. This duration was chosen based on empirical observations from our experimental system. Specifically, we consistently observed that females either laid eggs within 6 days after the blood meal or did not lay eggs at all. In addition, when females cannot access a sufficient protein intake to sustain oogenesis, egg development was initiated but subsequently followed by egg resorption a few days later. This pattern was observed in both parasitized and unparasitized mosquitoes. However, under the diluted blood condition, we observed a distinct response in parasitized females: they were able to develop several eggs, whereas in unparasitized females these developing eggs were systematically resorbed.

L430: Did the authors evaluate assumptions for their ANOVA? Some of the boxplots suggest distributions that may not be homoscedastic, but it is hard to tell from figures.

Model assumptions (e.g. normality of residuals, residual structure, heteroskedasticity and overdispersion) were assessed using the DHARMa package on residuals from the appropriate models (LM, LMM, GLM, or GLMM). This is now explicitly stated in the Methods section (Lines 518-519). When deviations from model assumptions were detected, data transformations or error distributions were adjusted accordingly. R scripts used for the analyses are now publicly available on Zenodo (https://zenodo.org/records/18374963).

"Model assumptions were assessed using graphical inspection and simulation-based residual diagnostics (*DHARMa*)."

4. **Journal Requirements and Technical Issues**

(i) An **Author Summary** has been added (lines 32-47).

"Mosquitoes host a wide diversity of microorganisms that can profoundly influence their biology, and their effects on mosquito physiology and reproduction are often more complex than expected. In this study, we examined the interaction between the Asian tiger mosquito *Aedes albopictus*, a major invasive disease vector, and its common gregarine parasite *Ascogregarina taiwanensis*. We show that this parasite spreads in two ways: unrelated interindividual transmission can occur through breeding sites, but its transmission is also biased toward the offspring of infected females because parasites are released into the water at egg-laying sites. Rather than acting solely as a cost to the host, infection was associated with changes in reproductive physiology: parasitized females produced larger eggs, laid them over a longer period, and gave rise to larger larvae, despite ingesting similar amounts of blood as uninfected females. Transcriptomic and physiological analyses indicate that these effects are linked to enhanced processing of blood-derived nutrients, particularly nitrogen, during oogenesis. These findings suggest that physiological responses to infection during reproduction can generate host benefits that also favor parasite transmission, illustrating how mutualistic traits may emerge as by-products of adaptation within predominantly parasitic interactions."

(ii) A complete list of **Supporting Information legends** has been added after the references.

(iii) All figures have been reformatted and uploaded as separate files following PLOS guidelines.

(iv) We confirm that all schematic elements in Figures 3, 4, 6, and S2 were drawn with the help of biorender. The Biorender Licence number was now added to the figure legends.

"Biorender was used to generate the figure (Licence number AR298GBO1A)."

(v) The **Financial Disclosure statement** has been rewritten in full sentences as requested.

5. Closing Statement

We again thank the Editor and Reviewers for their thoughtful and constructive feedback. We believe that the revisions have substantially strengthened the manuscript and clarified both the experimental design and the scope of our conclusions. We hope that the revised version will now be suitable for publication in *PLOS Pathogens*.
